# VDMNet: A Deep Learning Framework with Vessel Dynamic Convolution and Multi-Scale Fusion for Retinal Vessel Segmentation

**DOI:** 10.3390/bioengineering11121190

**Published:** 2024-11-25

**Authors:** Guiwen Xu, Tao Hu, Qinghua Zhang

**Affiliations:** 1 Department of Neurosurgery, Huazhong University of Science and Technology Union Shenzhen Hospital, Shenzhen 518052, China; virtuosoxu@163.com; 2 School of Information Science and Technology, Fudan University, Shanghai 200433, China; sitp6497@126.com

**Keywords:** retinal vessel segmentation, microvasculature structure, vessel dynamic convolution, multi-scale fusion

## Abstract

Retinal vessel segmentation is crucial for diagnosing and monitoring ophthalmic and systemic diseases. Optical Coherence Tomography Angiography (OCTA) enables detailed imaging of the retinal microvasculature, but existing methods for OCTA segmentation face significant limitations, such as susceptibility to noise, difficulty in handling class imbalance, and challenges in accurately segmenting complex vascular morphologies. In this study, we propose VDMNet, a novel segmentation network designed to overcome these challenges by integrating several advanced components. Firstly, we introduce the Fast Multi-Head Self-Attention (FastMHSA) module to effectively capture both global and local features, enhancing the network’s robustness against complex backgrounds and pathological interference. Secondly, the Vessel Dynamic Convolution (VDConv) module is designed to dynamically adapt to curved and crossing vessels, thereby improving the segmentation of complex morphologies. Furthermore, we employ the Multi-Scale Fusion (MSF) mechanism to aggregate features across multiple scales, enhancing the detection of fine vessels while maintaining vascular continuity. Finally, we propose Weighted Asymmetric Focal Tversky Loss (WAFT Loss) to address class imbalance issues, focusing on the accurate segmentation of small and difficult-to-detect vessels. The proposed framework was evaluated on the publicly available ROSE-1 and OCTA-3M datasets. Experimental results demonstrated that our model effectively preserved the edge information of tiny vessels and achieved state-of-the-art performance in retinal vessel segmentation across several evaluation metrics. These improvements highlight VDMNet’s superior ability to capture both fine vascular details and overall vessel connectivity, making it a robust solution for retinal vessel segmentation.

## 1. Introduction

Structural changes in the retinal microvasculature are closely associated with a range of ophthalmic and systemic diseases, including diabetic retinopathy, glaucoma, hypertensive retinopathy, and neurodegenerative conditions such as Alzheimer’s and Parkinson’s diseases [1,2,3]. Optical Coherence Tomography Angiography (OCTA), an emerging non-invasive and high-resolution imaging technique, enables detailed visualization of the retinal microvascular network, offering significant advantages over conventional fundus photography [4]. However, OCTA images are often affected by complex backgrounds, noise, and artifacts, which can hinder accurate vessel recognition [5,6]. Consequently, reliable retinal vessel segmentation is crucial for aiding diagnoses, monitoring disease progression, and evaluating treatment efficacy for these conditions [7]. Despite this importance, the task of retinal vessel segmentation remains challenging due to complex vascular structures, variability in image quality, and interference from pathological features.

The retinal vasculature consists of larger vessels, such as the central retinal artery and vein, and numerous smaller branches, including arterioles, venules, and capillaries, which have very fine diameters and are essential for nutrient and oxygen delivery to the retina [8]. Although these small vessels occupy a relatively small portion of the image, they are critical for accurate segmentation, as their omission or fragmentation can result in incomplete vessel maps [9]. Successful segmentation requires the extraction of these fine vessels to ensure vessel continuity and integrity. Moreover, retinal vessels exhibit significant variability in width along their paths, from the larger main trunks to the narrower peripheral branches, necessitating a model capable of accurately segmenting both wide and narrow vessels. The complexity is further compounded by the presence of bifurcations and crossings, which are particularly challenging for segmentation models to handle [10].

Early approaches to retinal vessel segmentation relied on traditional image processing algorithms, employing hand-crafted features and rule-based methods such as edge detection, morphological operations, and region growing techniques [11]. These methods, while useful in some contexts, were highly sensitive to image quality and often failed to capture complex vascular morphologies and finer vessel structures [12]. To overcome these limitations, more adaptive techniques, such as multi-scale filtering and local contrast enhancement, were introduced. For example, Liskowski and Krawiec proposed a multi-scale adaptive thresholding method that improved fine vessel detection [13]. However, these traditional methods still struggled to handle noise and vessel crossings, limiting their robustness [14]. With the advent of deep learning, particularly Convolutional Neural Networks (CNNs), the performance of retinal vessel segmentation has improved significantly. CNNs minimize the reliance on manual feature extraction by enabling end-to-end feature learning, which improves segmentation accuracy [15]. Advanced architectures such as UNet++ [16], Attention-UNet [17], and H-DenseUNet [18] further enhance the segmentation of fine structures by incorporating deeper networks and attention mechanisms. Despite these advancements, the local receptive fields of CNNs limit their ability to capture global features, which are critical for understanding the complex topology of retinal vascular networks [19]. To address this limitation, researchers have optimized loss functions, such as Dice loss, Tversky loss, and edge-aware losses, to mitigate class imbalance and enhance the retention of fine vessel details [20]. Generative Adversarial Networks (GANs) have also been applied to various aspects of medical image segmentation, including image enhancement, denoising, and data augmentation, contributing to improved segmentation robustness. For example, CycleGAN has been used for unsupervised domain adaptation, improving model generalization across different retinal imaging modalities [21]. GAN-based approaches have also been employed to enhance and denoise OCTA images, effectively reducing the impact of artifacts on segmentation [22]. Recent advancements in Self-Supervised Learning (SSL) and few-shot learning have further improved medical image segmentation. SSL leverages large-scale unannotated data for pre-training, enhancing performance when labeled data are scarce [23]. Studies in 2022 demonstrated that SSL significantly improved detail retention and consistency in OCTA image segmentation [24]. Few-shot learning, particularly meta-learning, has shown promise in addressing data scarcity by enabling rapid adaptation to small datasets and improving segmentation outcomes [25].

OCTA’s ability to visualize the retinal microvasculature with high precision has sparked significant interest in retinal vessel segmentation. However, OCTA images often contain substantial noise and artifacts that complicate the segmentation task [26]. To address these challenges, recent methods have incorporated multi-scale feature fusion and denoising techniques, such as MS-Net [27] and Hybrid ResUNet [28], to segment vascular structures across different scales and mitigate the impact of noise. MAGRes-UNet incorporates attention gates into the skip connections to emphasize relevant features and suppress noisy or irrelevant responses [29]. Integrating image enhancement with model optimization has further improved the robustness of OCTA image segmentation [30]. The emergence of transformers, with their global receptive fields and attention mechanisms, offers new opportunities for handling the complex structures of vascular networks. TransUNet [31], which combines the strengths of transformers and UNet, strikes a balance between global feature extraction and detailed segmentation. The Swin Transformer [32], with its sliding window approach, enhances the retention of fine details and overall vascular connectivity, significantly improving segmentation quality. Hybrid models, such as Swin-UNet [33] and SegFormer [34], that integrate CNNs’ local feature learning with transformers’ global feature extraction, have shown superior performance in retinal vessel segmentation [35].

Despite these advances, challenges remain. Accurately segmenting crossing and overlapping vessels, preserving fine terminal branches, and dealing with noise, low contrast, and uneven illumination remain persistent difficulties. Key challenges include the following: (1) handling complex vascular morphologies, such as curved, crossing, and fine branches, which complicate segmentation; (2) addressing multi-scale vessel segmentation, where both large main vessels and small capillaries need to be segmented accurately despite scale variations; (3) overcoming low contrast and noise, which obscure vessel boundaries; and (4) maintaining microvascular connectivity and detail retention, as many models struggle to preserve the continuity and fine details of small vessels. Additionally, interference from high-intensity regions, such as the optic disc and pathological areas like exudates and hemorrhages, further complicates segmentation tasks.

To address these challenges, this paper proposes VDMNet, a novel retinal vessel segmentation network that incorporates cutting-edge techniques, including Fast Multi-Head Self-Attention (FastMHSA), Vessel Dynamic Convolution (VDConv), Multi-Scale Fusion (MSF), and Weighted Asymmetric Focal Tversky Loss (WAFT Loss), to enhance segmentation accuracy and robustness. Specifically, the main contributions of this work are as follows:

1. We propose the FastMHSA module, which adaptively focuses on both local and global information across different regions of the image, improving the model’s robustness against complex backgrounds and pathological interference.
2. We propose the VDConv structure to enhance the model’s adaptability to vascular morphology, particularly for curved and crossing microvessels, significantly improving segmentation accuracy.
3. We design the MSF mechanism to capture both global and local vessel information by fusing features across multiple scales, enhancing the capture of fine vessels and improving vessel connectivity and detail retention.
4. We employ WAFT Loss, a weighted asymmetric loss function, to address class imbalance in retinal vessel segmentation, enhancing the identification of small and difficult-to-segment vessels.

## 2. Materials and Methods

### 2.1. Datasets

To evaluate the performance of our proposed network, we utilized two widely used public datasets: ROSE-1 and OCTA-500 (specifically, the OCTA-3M subset), both of which include key vascular structures, such as arteries, veins, and capillaries, across the retinal layers. In the publicly available datasets we used, the 3D OCTA volumes were pre-processed by the dataset providers to generate 2D representations. Specifically, these 2D images, often referred to as en face angiograms, were derived by projecting specific layers or regions of interest from the original 3D volume into a 2D view using projection methods such as maximum or average projection, and subsequently compiled into a 2D dataset.

#### 2.1.1. ROSE-1

The ROSE-1 dataset, provided by the Cixi Institute of Biomedical Engineering, Ningbo Institute of Industrial Technology, Chinese Academy of Sciences, consists of 117 OCTA images from 39 subjects. This dataset includes 26 individuals diagnosed with Alzheimer’s disease (AD) and 13 healthy controls. All scans were captured using the RTVue XR Avanti SD-OCT system (Optovue, Fremont, CA, USA), equipped with the AngioVue software (version 2015.1.0.90). The images have a resolution of 304 × 304 pixels, covering a 3 × 3 mm^2^ area centered on the fovea, specifically within an annular zone ranging from 0.6 mm to 2.5 mm in diameter around the foveal center [36]. For our study, we selected en face angiograms of the superficial vascular complex (SVC) with corresponding pixel-wise ground truth annotations (39 images). The dataset was split into three sets: a training set (27 images), a validation set (3 images), and a testing set (9 images).

#### 2.1.2. OCTA-500

The OCTA-500 dataset, provided by the School of Computer Science and Engineering at Nanjing University of Science and Technology, comprises two subsets: OCTA-6M, which contains images from 300 subjects with a 6 mm × 6 mm field of view (FOV), and OCTA-3M, which includes data from 200 subjects with a 3 mm × 3 mm FOV. The images were acquired using a commercial 70 kHz spectral-domain OCT system with a center wavelength of 840 nm (RTVue-XR, Optovue, CA, USA) [37,38]. For our study, we utilized the OCTA-3M subset, specifically focusing on the inner limiting membrane to the outer plexiform layer (ILM-OPL) region using the maximum projection. This subset was divided into three sets: a training set (140 images), a validation set (10 images), and a testing set (50 images).

### 2.2. Network Architecture

**Figure 1:**
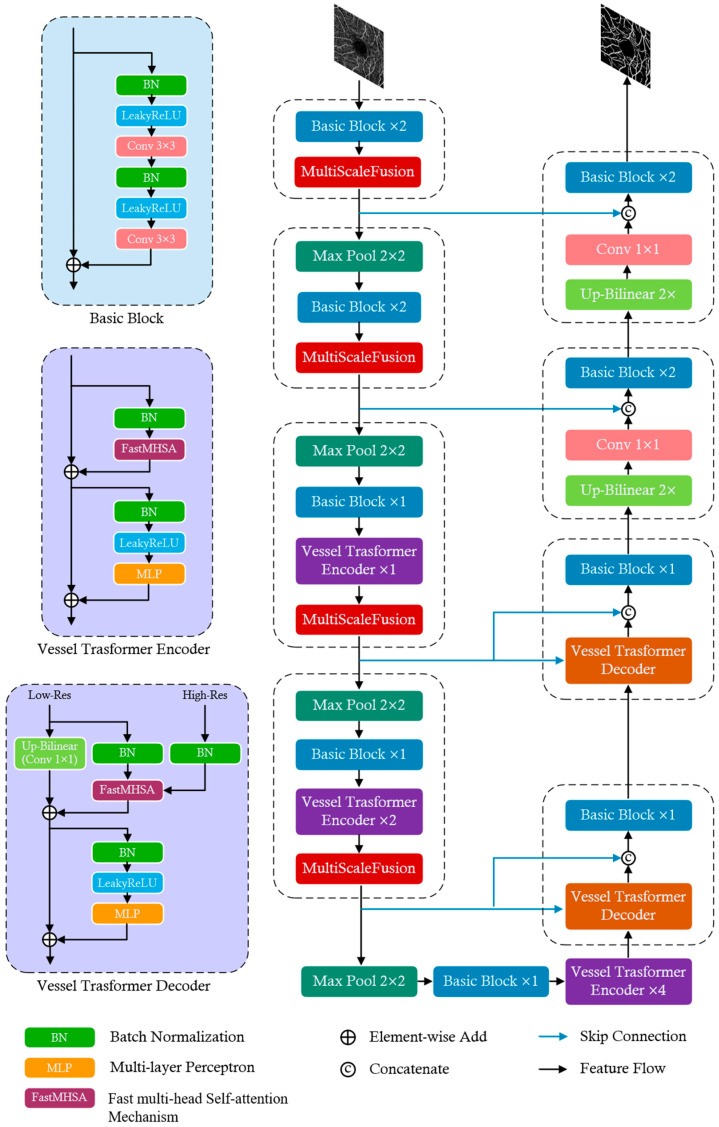
The architecture of VDMNet, which is composed of encoder, decoder, and skip connections.

The architecture of the proposed VDMNet is illustrated in Figure 1. This network combines CNN components with transformer encoders and decoders, structured within a U-Net-like framework to achieve effective retinal vessel segmentation.

The encoder section comprises multiple Basic Blocks, MSF modules, Max Pooling layers, and Vessel Transformer Encoders. Each Basic Block consists of two sets of convolutional layers, as well as batch normalization and LeakyReLU activation functions integrated with residual connections to increase network depth and mitigate the vanishing gradient problem. The inclusion of the MSF module during encoding allows the network to effectively capture vascular features across different scales, enhancing the recognition of fine vessels and bifurcations. At deeper levels of the encoder, Vessel Transformer Encoder modules are introduced. Each Vessel Transformer Encoder includes batch normalization, FastMHSA, LeakyReLU activation, and a multi-layer perceptron (MLP). This design leverages the global receptive field of transformers, compensating for the limitations of traditional convolutional networks in capturing global context, thus improving the modeling of complex vascular structures.

The decoder section consists of multiple decoding modules, including Vessel Transformer Decoders, Basic Blocks, Up-Conv layers, and feature fusion operations. Corresponding to the Vessel Transformer Encoders in the encoder section, Vessel Transformer Decoders are employed during the decoding phase and integrated with skip connections to merge high-level global information with low-level local features. This approach enables a more precise reconstruction of vascular details, enhancing segmentation accuracy and connectivity. The decoder progressively restores the spatial resolution of the feature maps through the Up-Conv layers and incorporates skip connections to utilize the detailed information from the encoder. This ensures efficient feature propagation and the accurate reconstruction of the vascular morphology, resulting in precise retinal vessel segmentation.

### 2.3. The Proposed Fast Multi-Head Self-Attention Mechanism

As images are highly structured data, most pixels in high-resolution feature maps within local footprints share similar features, except for in the boundary regions. Therefore, the pair-wise attention computation among all pixels is highly inefficient and redundant; self-attention is essentially low rank for long sequences, which indicates that most information is concentrated in the largest singular values. The main idea is to use two projections to project the keys and values: $K,V\in R^{n\times d}$ into low-dimensional embedding, and $\bar{K},\bar{V}\in R^{k\times d}$, where k=hw≪n,h and w is the reduced size of the feature map after sub-sampling. By doing so, the computational complexity is reduced to $O\left(nkd\right)$ [39].

(1) $$Attention\left(Q,\bar{K},\bar{V}\right)=\underset{\bar{P}:n\times k}{\underbrace{\mathrm{softmax}\left(\frac{Q{\bar{K}}^{T}}{\sqrt{d}}\right)}}\underset{k\times d}{\underbrace{\bar{V}}}$$

Retinal vessels are characterized by their elongated and thin structures, where most pixels belong to the background, and the target pixels representing vessels are sparse, primarily concentrated along vessel edges. Effective retinal vessel segmentation requires the model to capture both fine-grained local details, such as small vessels and bifurcations, and the broader global vascular context. Traditional self-attention mechanisms are powerful tools for capturing global dependencies, but their computational complexity becomes a limiting factor when applied to high-resolution images, such as retinal scans, or to sequences of long-range dependencies.

**Figure 2:**
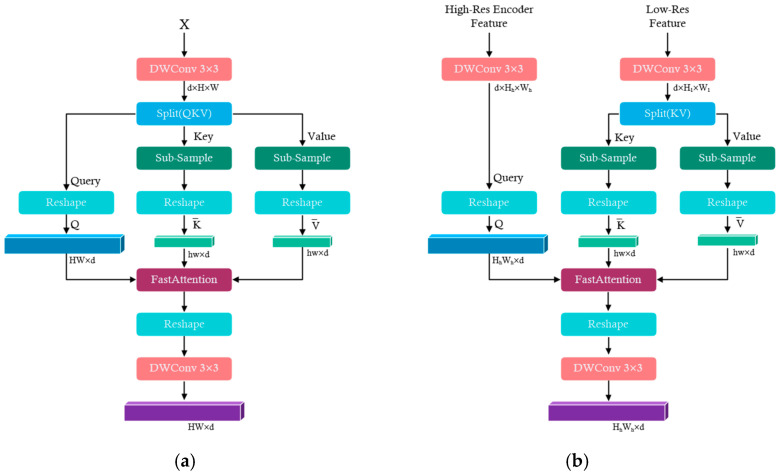
The proposed Fast Multi-Head Self-Attention Mechanism. (**a**) Fast Multi-Head Self-Attention Mechanism encoder. (**b**) Fast Multi-Head Self-Attention Mechanism decoder. They share similar concepts, but (**b**) takes two inputs: the high-resolution features from skip connections in the encoder and the low-resolution features from the decoder.

The FastMHSA module, depicted in Figure 2, addresses these computational challenges by employing a dimensionality reduction strategy for the keys (*K*) and values (*V*) while preserving the quality of attention. This reduction decreases the overall computational cost associated with self-attention. However, such dimensionality reduction can introduce potential information loss, especially in the context of detailed medical images like OCTA scans. To mitigate this issue, FastMHSA uses random feature decomposition to approximate full attention, maintaining computational efficiency while minimizing information loss. This technique enables the network to retain the expressive power of full self-attention without incurring prohibitive memory and computation costs. By leveraging random feature approximation, FastMHSA efficiently captures both local details (e.g., small vessels and bifurcation points) and global structures (e.g., overall vascular layout), ensuring that even complex regions like vessel crossings are accurately segmented.

As shown in Figure 2a, the encoder-side FastMHSA processes the input features through Depthwise Separable Convolution (DWConv) to capture vascular structures efficiently. The features are then split into query (*Q*), key (*K*), and value (*V*), followed by sub-sampling to reduce the dimensions of the keys and values. These are passed through the Fast Attention module, where approximate attention scores are computed. The output is reshaped and refined with a 3 × 3 depthwise separable convolution layer before further processing. In the decoder, as depicted in Figure 2b, high-resolution and low-resolution features are combined. These features are processed by DWConv and split into keys and values, which are sub-sampled and passed through the Fast Attention module to aggregate both local and global information. The result is reshaped and processed through a 3 × 3 depthwise separable convolution to prepare the features for segmentation.

By integrating FastMHSA within VDMNet, the network can efficiently capture both microvascular structures and broader vascular patterns, improving overall segmentation performance in noisy, low-contrast conditions.

### 2.4. Vessel Dynamic Convolution

In the proposed retinal vessel segmentation network, we introduce a novel convolutional module termed VDConv, which replaces the upper DWConv used for input feature processing in the FastMHSA module. This module is specifically designed to address critical challenges in retinal vessel segmentation, including the extraction of fine vessels, handling complex vascular morphologies (such as curvature variations, width changes, bifurcations, and crossings), and mitigating issues related to low contrast and noise in images. VDConv dynamically adjusts the shape and size of convolutional kernels, allowing the model to adaptively capture intricate vessel structures, thereby enhancing segmentation accuracy and robustness.

VDConv is composed of the following key components:

1. Offset Prediction and Feature Interpolation: The offset prediction module dynamically predicts and adjusts the positions of convolutional kernels, enabling the convolution paths to adaptively align with vessel structures. Through feature interpolation, VDConv effectively captures fine and curved vessels, improving segmentation performance in complex morphologies.
2. Dynamic Weight Generator: This component leverages convolutional layers to generate and normalize the weights of a set of expert groups. By dynamically adjusting the contribution of each group, this mechanism enhances the model’s ability to handle challenging scenarios such as complex backgrounds and class imbalances, thereby improving segmentation robustness and flexibility.
3. Multi-Expert Convolution: VDConv employs a multi-expert convolution strategy, where each expert group focuses on different feature directions and scales, implemented via group convolution. The dynamically generated weights control the contribution of each expert group, allowing the model to better manage vessel crossings and fine branches, effectively reducing breakages and preserving detailed vessel structures.

The VDConv operation can be mathematically formulated as follows:

- Offset Prediction: Given an input feature map $X\in R^{C\times H\times W}$, the offset prediction module computes offsets
  (2) $$O=tanh(GN\left({Conv}_{offset}\left(X\right)\right))\in R^{2K\times H\times W}$$
  where ${Conv}_{offset}$ is a convolutional layer predicting the offsets for K kernel positions, and tanh normalizes the offset values within [−1, 1].
- Deformable Convolution: The deformable convolution is applied using the following predicted offsets:
  (3) $$Y_{i,j}=\sum_{k=1}^{K}W_{k}\cdot X\left(i+O_{i}^{y},j+O_{j}^{x}\right)$$
  where $W_{k}$ represents the kernel weights and $O_{i}^{y},O_{j}^{x}$ are the predicted offsets for the k-th position.
- Multi-Expert Fusion: The output from the deformable convolutions is processed by multiple expert kernels, and their outputs are fused as follows:
  (4) $$Z=\sum_{n=1}^{N}\alpha_{n}\cdot Y_{n}$$
  where $\alpha_{n}=$ softmax$\left(W_{gen}\right)$ is the dynamically generated weight for N experts from the weight generator, ensuring that the model adapts the fusion of features based on input content.

### 2.5. Multi-Scale Fusion

Retinal vessels exhibit diverse scales and morphologies, ranging from large primary vessels to fine capillaries, with varying densities across different regions. Traditional single-scale methods often miss fine vessel details during downsampling or feature extraction, leading to incomplete segmentation. To address this, the MSF module enhances the model’s ability to extract detailed information from high-resolution feature maps, improving the accuracy of fine vessel segmentation and preventing the loss of critical structures during downsampling [27,40]. By facilitating information sharing between low-level detailed features and high-level abstract features, the MSF module enriches feature representation, enabling the model to capture both global vessel structures and intricate details.

**Figure 3:**
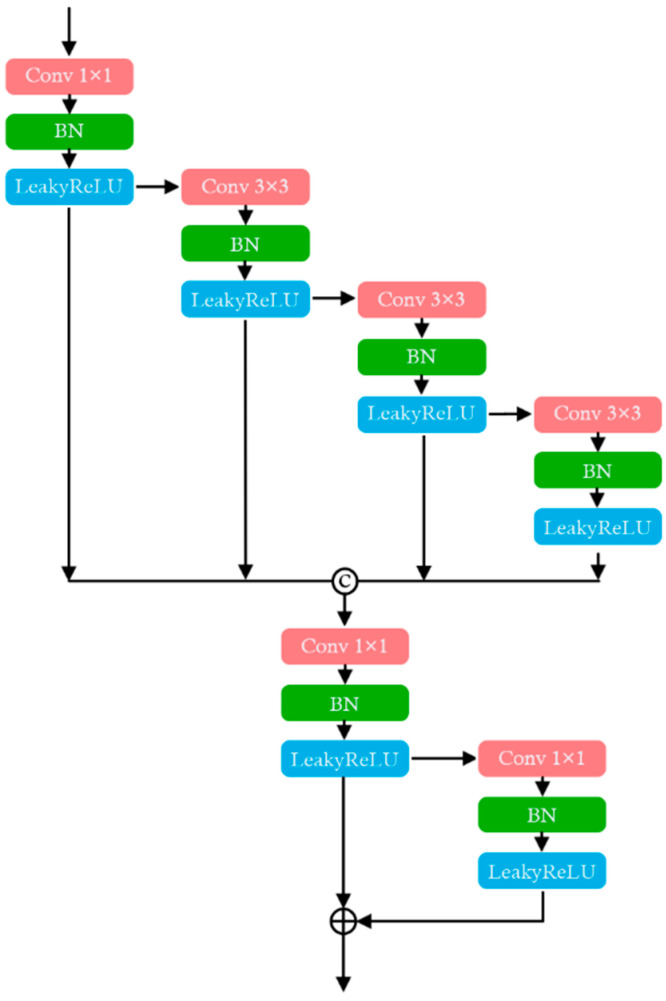
Multi-Scale Fusion Module.

As shown in Figure 3, the MSF module improves feature representation through multi-scale convolutional operations, designed specifically to capture fine and complex vessel segments. It employs convolutional kernels at different scales to achieve comprehensive feature extraction: a 1 × 1 convolution adjusts the channel dimensions while preserving spatial information, and a 3 × 3 convolution with Standard Padding captures local spatial characteristics. To expand the receptive field, 3 × 3 convolutions with dilation = 2 and dilation = 3 are used, allowing the model to capture both broader structures and elongated vessel patterns.

Each convolutional layer is followed by batch normalization (BN) and LeakyReLU activation, which stabilize the feature distributions and introduce non-linearity to enhance model learning. The outputs from these layers are concatenated along the channel dimension to fuse multi-scale features, ensuring the capture of both fine details and broader vessel structures. To compress the concatenated features and reduce redundancy, a final 1 × 1 convolution is applied. Additionally, residual connections help to optimize feature fusion, mitigating the vanishing gradient problem and enhancing the robustness of feature learning.

Within the VDMNet architecture, the MSF modules are applied at strategic points in the encoder, following key feature extraction stages such as the Basic Block and Vessel Transformer Encoder stages. By fusing multi-scale information before downsampling, the MSF module helps preserve fine vessel details that would otherwise be lost, thus maintaining vascular continuity and improving the segmentation of delicate vessel structures.

This strategic use of the MSF module ensures that both fine local details and larger vessel structures are effectively captured. The integration of dilated convolutions further extends the receptive field, allowing the network to capture elongated vessel patterns while maintaining fine detail recognition. By enhancing feature extraction at multiple levels, the MSF module significantly improves the segmentation accuracy of fine vessels, contributing to the overall robustness and precision of the VDMNet model in retinal vessel segmentation tasks.

### 2.6. Weighted Asymmetric Focal Tversky Loss

In retinal vessel segmentation, a major challenge is the inherent imbalance between the foreground (vessels) and the background. Vessels often occupy only a small portion of the image, and the fine, intricate nature of capillaries makes them difficult to segment accurately. Additionally, noise, artifacts, and variability in vessel morphology further complicate segmentation. To address these issues, we employ WAFT Loss, which is specifically designed to manage class imbalance, emphasize difficult-to-segment regions, and ensure the robust segmentation of intricate structures.

False positives (FPs) can lead to the overestimation of the vascular network, misdiagnosis, or unnecessary treatments, while false negatives (FNs) can result in missed critical vascular structures, delaying necessary treatment. WAFT Loss builds upon the Tversky Index, a generalization of the Dice coefficient, allowing for flexible control over both FPs and FNs. This flexibility is crucial for retinal vessel segmentation, where minimizing FNs (missed vessels) is often prioritized, but controlling FPs (misclassified background) remains important. By adjusting the weight parameter *δ*, WAFT Loss effectively reduces FN without excessively penalizing FPs, maintaining a balance that is critical for preserving the continuity of fine vessel structures, which are prone to omission in standard segmentation tasks.

To further enhance its flexibility, WAFT Loss introduces two weight parameters, α and β, which control the relative importance of the background (non-vessel areas) and foreground (vessels), respectively. Assigning a higher weight to the foreground class allows the model to focus more on vessels, especially the fine and delicate structures that are often under-segmented. We used α = 0.2 and β = 0.8, and these values can be adjusted depending on the dataset’s background-to-foreground ratio. These weights provide an additional level of customization, enabling the loss function to be tailored to the specific segmentation task or dataset characteristics.

The Tversky Index (*TI*) is defined as follows:

(5) $$TI=\frac{TP+\epsilon }{TP+\delta \cdot FN+\left(1-\delta \right)\cdot FP+\epsilon }$$

where

- *TP* represents true positives;
- *FN* represents false negatives;
- *FP* represents false positives;
- *δ* is a coefficient that controls the weights of false positives and false negatives;
- *ϵ* is a small constant introduced to prevent division by zero and ensure numerical stability during training.

WAFT Loss incorporates a focal adjustment that down-weights easy-to-segment regions, emphasizing challenging areas to force the model to focus on fine vessels or complex intersections. This is achieved by applying the focal term ${\left(1-{TI}_{fg}\right)}^{-\gamma }$ to the foreground Tversky Index, which magnifies the contribution of difficult-to-segment regions.

The final WAFT Loss function is expressed as follows:

(6) $$L_{WAFT}=\alpha \cdot \left(1-{TI}_{bg}\right)+\beta \cdot \left[\left(1-{TI}_{fg}\right)\cdot {\left(1-{TI}_{fg}\right)}^{-\gamma }\right]$$

where

- $L_{WAFT}$ is WAFT Loss;
- $TI_{bg}$ and *T*$I_{fg}$ denote the Tversky Index for the background and foreground (vessels) classes, respectively;
- α and β are the weights for background and foreground, controlling the importance of different classes;
- γ is the focal parameter that enhances the model’s focus on difficult-to-classify examples.

In retinal vessel segmentation, the fine, elongated nature of capillaries, combined with the variability in vessel width and branching, makes accurate segmentation particularly challenging. WAFT Loss plays a crucial role in ensuring that the model can segment both large vessels and small capillaries. By providing precise control over the trade-off between false positives and false negatives, as well as between the background and foreground, WAFT Loss ensures the maintenance of vessel connectivity and minimizes false classifications. This balance is critical for accurately segmenting retinal vessels and preserving fine structural details.

## 3. Experiment and Results

### 3.1. Implementation Details

The proposed VDMNet is implemented using the PyTorch framework. All networks are trained for 100 epochs with a batch size of 1. The training process uses the Adam optimizer with a momentum of 0.9 and a weight decay of 0.001. A polynomial learning rate schedule is applied, following the formula $lr=base\_lr{\left(1-iter/\left(max\_iter\right)\right)}^{power}$. Data augmentation techniques include random left–right flipping, top–down flipping, and rotations ranging from −10° to 10°.

### 3.2. Evaluation Indicators

We evaluated the segmentation performance of our proposed network using the following metrics: the Dice Coefficient (DICE), the Jaccard Index (JAC), Balanced Accuracy (BACC), Recall (REC), the Connectivity Area Length Metric (CAL), and the Largest Connected Component Ratio (LCC).

Dice Coefficient (*DICE*): This metric measures the similarity between two samples. The formula is

(7) $$DICE=\frac{2\times TP}{2\times TP+FP+FN}$$

Jaccard Index (*JAC*): This metric calculates the similarity between two sets. The formula is

(8) $$JAC=\frac{TP}{TP+FP+FN}$$

Balanced Accuracy (*BACC*): This metric is the average of sensitivity and specificity and is used to address class imbalance issues. The formula is

(9) $$BACC=\frac{1}{2}\left(\frac{TP}{TP+FN}+\frac{TN}{TN+FP}\right)$$

Recall (*REC*): This metric represents the proportion of actual positive samples that are correctly predicted as positive. The formula is

(10) $$REC=\frac{TP}{TP+FN}$$

where *TP* and *FP* represent true positives and false positives, respectively, and *TN* and *FN* represent true negatives and false negatives, respectively.

Furthermore, for the evaluation of the global quality of segmentation, we used the Connectivity Area Length (*CAL*) metric proposed by Gegundez-Arias [41]. It is based on three descriptive features:

Connectivity (*C*), to assess the fragmentation degree between segmentations, described mathematically by the following formula:

(11) $$C\left(S,S_{GT}\right)=1-min\left(1,\frac{\left|{\#}_{C}S_{GT}-{\#}_{C}S\right|}{\#S_{GT}}\right)$$

where ${\#}_{C}S$ and ${\#}_{C}S_{GT}$ represent the number of connected components in the segmented and ground truth image, while $\#S_{GT}$ is the number of vessel pixels in the mask.

Area (*A*), to evaluate the degree of overlapping, defined as

(12) $$A\left(S,S_{GT}\right)=\frac{\#(\left(\delta_{\alpha }\left(S\right)\cap S_{GT}\right)\cup \left(S\cap \delta_{\alpha }\left(S_{GT}\right)\right)}{\#\left(S\cup S_{GT}\right)}$$

where $\delta_{\alpha }$ represents a morphological dilation using a disc of radius α.

Length (*L*), to capture the degree of coincidence, described by

(13) $$L\left(S,S_{GT}\right)=\frac{\#\left(\left(\varphi \left(S\right)\cap \delta_{\beta }S_{GT}\right)\right)\cup \left(\delta_{\beta }\left(S\right)\cap \varphi \left(S_{GT}\right)\right)}{\#\left(\varphi \left(S\right)\cup \varphi \left(S_{GT}\right)\right)}$$

where φ indicates a skeletonisation procedure and $\delta_{\beta }$ is a morphological dilation using a disc of radius β.

The *CAL* metric is defined as follows:

(14) $$CAL\left(S,G\right)=C\times A\times L$$

Considering vessel width and the closeness between capillaries in OCTA images, we set both α and β to equal 1. The product of the *C*, *A*, and *L* (*CAL*) results is sensitive to the vascular features and takes values in the range of [0, 100], with 0 denoting the worst segmentation and 100 representing the perfect segmentation.

We introduce the Largest Connected Component Ratio (*LCC*) [41] with the aim of penalizing those methods that do not retrieve connections of the vascular network. *LCC* is defined as

(15) $$LCC=1-min\left(1,\frac{\left|\#LCC_{S}-\#LCC_{GT}\right|}{\#LCC_{GT}}\right)$$

where $\#LCC_{S}$ and $\#LCC_{GT}$ represent the lengths, in terms of number of pixels, of the largest connected component in the skeleton of the segmented and ground truth images.

The closer the *LCC* ratio is to 100, the more structurally similar the largest connected component of the segmented image is to the ground truth.

### 3.3. Ablation Experiments

We conducted an ablation study on the ROSE-1 dataset to assess the impact of each key component—FastMHSA, VDConv, and MSF—by progressively integrating them into the baseline model. The results, as shown in Table 1, demonstrate the complementary contributions of these modules, culminating in the best performance when all are combined.

Starting from the baseline model, which achieved a DICE score of 87.63 and a JAC of 77.99, we observed moderate improvements when either FastMHSA or VDConv was introduced. FastMHSA enhanced the model’s ability to focus on important vessel regions, improving the DICE score to 87.93, while VDConv, which adapts to complex vessel morphologies, achieved similar gains with a DICE score of 87.87. The most significant improvement came with the addition of the MSF module, which raised the DICE score to 88.57. MSF’s ability to capture both fine details and larger vessel structures proved critical in improving segmentation accuracy, particularly for small vessels that are often missed by traditional methods. This demonstrates that multi-scale feature aggregation is essential for precise retinal vessel segmentation. However, the individual or partial combination of components resulted in incremental improvements, indicating that these modules alone only partially addressed the challenges of fine vessel segmentation and maintaining continuity.

When we combined multiple modules, their synergistic effects became apparent. The joint integration of FastMHSA, VDConv, and MSF led to the best results, with a DICE score of 89.36 and a JAC of 80.79. This substantial improvement highlights the complementary nature of these components: FastMHSA enhances global contextual awareness, VDConv improves vessel morphology adaptation, and MSF ensures multi-scale consistency. Together, they allow the model to balance fine vessel segmentation with overall vascular structure preservation, significantly boosting overall performance.

In summary, the ablation study confirms that each module contributes uniquely to improving segmentation, but their combined effect is much greater, leading to state-of-the-art performance in retinal vessel segmentation.

To further understand the effectiveness of various loss functions on the proposed framework, we replaced our proposed WAFT Loss with other commonly used loss functions and conducted a comparative study on the ROSE-1 dataset to evaluate their impact on segmentation performance. The loss functions tested included Dice Loss, Cross Entropy Loss, Tversky Loss, Focal Loss, Asymmetric Focal Tversky Loss (AFT Loss), and WAFT Loss. The evaluation was based on key metrics such as DICE, JAC, BACC, REC, CAL, and LCC, as summarized in Table 2.

The proposed WAFT Loss outperformed other loss functions across almost all metrics, achieving the highest DICE (89.36) and JAC (80.79) scores, indicating superior overlap and fine vessel segmentation. Additionally, WAFT Loss led to the best BACC (94.32) and REC (90.63), which are critical for correctly identifying vessels and balancing predictions between foreground and background classes. These results clearly demonstrate the effectiveness of WAFT Loss in improving segmentation performance, particularly for complex vessel structures.

Our comparison of loss functions reveals that each approach has its strengths but also distinct limitations when applied to retinal vessel segmentation. Dice Loss and Cross Entropy Loss, while effective for balanced datasets, struggle with the inherent class imbalance in OCTA images, particularly in preserving fine vessels, as reflected in their lower CAL and JAC scores. Tversky Loss, which introduces a trade-off between false positives and false negatives, partially mitigates this issue, improving balance in predictions, but still falls short in maintaining vessel continuity. Focal Loss enhances segmentation in hard-to-classify areas, leading to better REC, but tends to over-emphasize these regions at the cost of global structure, as indicated by its lower JAC and LCC scores. AFT Loss, by integrating focal elements into Tversky Loss, further refines this balance, but its ability to maintain global vascular continuity remains limited, indicating that while it effectively addresses class imbalance, it struggles to preserve the entire vascular network’s structural integrity. These results highlight the need for a loss function that not only balances class predictions, but also ensures both local detail segmentation and global vessel continuity—challenges that WAFT Loss was specifically designed to address.

Compared to AFT Loss, the key improvement in WAFT Loss lies in the introduction of explicit weights to the background and foreground classes, allowing for better control over their importance. By assigning a higher weight to the foreground (vessels) and a lower weight to the background, WAFT Loss ensures the model focuses more on correctly identifying vessels, particularly smaller and harder-to-segment structures. This targeted focus is critical in retinal vessel segmentation, where missing even small vessels can have significant implications.

The introduction of these class-specific weights further mitigates the impact of class imbalance, allowing WAFT Loss to dynamically balance these factors and thereby enhance overall segmentation performance, as reflected in its higher CAL and LCC scores. By prioritizing vessel detection and ensuring structural continuity, WAFT Loss improves the model’s ability to capture smaller, harder-to-segment vessels, which in turn enhances the continuity and connectivity of the overall vessel network. This improvement is reflected in the higher DICE and CAL scores, as the segmented vessels are more likely to form larger connected components and maintain structural continuity across the entire retinal image.

### 3.4. Comparison Experiments

We evaluated VDMNet on two publicly available OCTA datasets, ROSE-1 and OCTA-3M, comparing it against several state-of-the-art segmentation models, which we refer to as compared networks, including three convolution-based networks (UNet, UNet3+, and VesselNet), two transformer-based networks (SwinUNet and SegFormer), and two hybrid transformer networks (TransUNet and UTNet). The results for these comparison methods, as reported in [42], evaluated under consistent experimental settings, serve as a benchmark. All models were assessed using key metrics such as DICE, JAC, BACC, REC, CAL, and LCC, as summarized in Table 3 and Table 4.

VDMNet consistently delivered superior performance across almost all metrics. On the ROSE-1 dataset, VDMNet achieved the highest DICE (89.36) and JAC (80.79) scores, surpassing all competing models. Similarly, on the OCTA-3M dataset, VDMNet’s DICE score reached 91.66, with a JAC score of 84.67, showcasing its robust segmentation capability. Additionally, VDMNet excelled in CAL (87.28) and LCC (89.21) on OCTA-3M, demonstrating its strength in preserving vessel continuity and correctly identifying fine vessels. VDMNet also showed competitive performance in terms of BACC and REC, highlighting its balanced handling of both the foreground (vessels) and background regions, while producing reliable probability estimates. These results suggest that VDMNet is effective in capturing both fine vascular structures and overall connectivity.

Convolution-based networks like UNet, UNet3+, and VesselNet have long been the backbone of medical image segmentation tasks. These models excelled in local feature extraction, as reflected in their relatively strong DICE and REC scores. VesselNet, for example, achieved a DICE score of 86.19 on ROSE-1 and 90.90 on OCTA-3M, indicating its proficiency in capturing finer details. However, these networks struggled with maintaining global vessel continuity, as seen in their lower CAL and LCC scores. This limitation suggests that, while convolutional models are strong in detailed local feature extraction, they often miss the global context necessary for handling complex retinal vessel structures.

With the rise of transformer-based models, which focus on capturing global context and long-range dependencies, there was hope to address these limitations. However, as demonstrated by SwinUNet and SegFormer’s lower scores, particularly on the ROSE-1 dataset, the pure transformer models did not perform as well as expected in retinal vessel segmentation. SwinUNet’s lower DICE and JAC scores suggest that while transformers capture broader context, they lack the ability to handle fine-grained structures such as small vessels. SegFormer showed some improvement over SwinUNet, showing higher DICE and JAC scores on the OCTA-3M dataset in particular, while performing similarly on the ROSE-1 dataset, but the extent of improvement remained relatively limited. Furthermore, their combined performance in terms of CAL and LCC revealed that transformer-based models struggled to maintain vessel continuity and connectivity, likely due to their limited local feature extraction capabilities.

In contrast, hybrid models like TransUNet and UTNet, which combine convolutional layers with transformer components, demonstrated a better balance between local and global feature extraction. TransUNet showed moderate performance, with a relative balance across various metrics on both datasets, reflecting some improvements over traditional models but still falling short in overall accuracy. Its LCC score on ROSE-1 indicates TransUNet’s capability in maintaining vessel continuity, although it was not as effective as UTNet in terms of combined CAL and LCC scores. UTNet, on the other hand, performed competitively, especially in terms of BACC and REC on the OCTA-3M dataset, demonstrating its superior ability to accurately segment and detect vessels compared to both TransUNet and purely convolution-based models. Despite this balanced approach, UTNet still fell short of VDMNet in overall segmentation accuracy, as reflected by its lower DICE and JAC scores. This result highlights that, while hybrid models like TransUNet and UTNet offer improvements over traditional convolutional and transformer-based architectures, further integration of advanced modules, as seen in VDMNet, remains crucial for optimal performance.

VDMNet integrates the strengths of both convolutional and transformer architectures, providing a well-balanced approach to retinal vessel segmentation. By incorporating advanced components like FastMHSA, VDConv, and MSF, VDMNet is able to capture both fine details and larger vessel structures. This integration ensures superior performance across key metrics, including DICE, JAC, REC, and LCC, demonstrating VDMNet’s effectiveness in both local and global feature extraction.

The comparison experiments illustrate that while convolution-based networks are adept at extracting local details and transformer-based models excel at capturing global context, VDMNet achieves the best of both worlds by integrating these two approaches. Its hybrid architecture consistently outperforms other models across a range of key metrics, making it particularly effective for retinal vessel segmentation tasks. VDMNet’s ability to maintain both fine vessel segmentation and overall structural continuity positions it as a state-of-the-art solution for OCTA image analysis.

**Figure 4:**
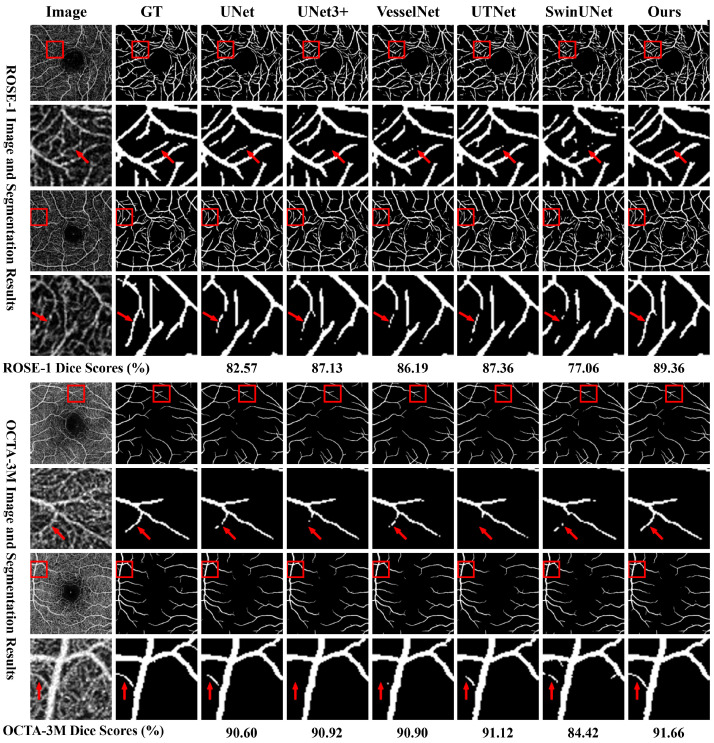
Retinal vessel segmentation results of the proposed VDMNet and other segmentation networks. From top to bottom, the OCTA images of rows 1 and 3 come from ROSE-1, and rows 5 and 7 come from OCTA-3M, respectively. Rows 2, 4, 6, and 8 show the corresponding locally zoomed-in OCTA images, as well as the ground truth and segmentation results.

We conducted our evaluation of VDMNet against several state-of-the-art models, including UNet, UNet3+, VesselNet, UTNet, and SwinUNet, under consistent experimental settings, as shown in Figure 4. The segmentation results were generated using the OCTA images from both the ROSE-1 and OCTA-3M datasets. These results highlight VDMNet’s superior ability to capture fine vessel structures, maintain vascular connectivity, and handle complex vessel morphologies.

One of the key challenges in retinal vessel segmentation is accurately detecting small, thin vessels, which are often missed by many models. As seen in the zoomed-in regions of Figure 4 (rows 2 and 4), models like VesselNet and SwinUNet struggle to detect these finer structures, particularly around bifurcations. While UNet and UNet3+ performed better, some smaller vessels still remained fragmented or were missing endpoints. In contrast, VDMNet, utilizing the MSF module, effectively captures these fine vessels by fusing global and local features across multiple scales, ensuring vessel continuity and integrity.

Each model has its unique approach to handling segmentation; for example, UTNet shows some proficiency in dealing with fine structures. However, due to a lack of high-resolution global information, false positives remain inevitable. Maintaining the overall vascular structure, particularly at bifurcations and crossing points, is also crucial for accurate segmentation. The FastMHSA module in VDMNet enhances the model’s ability to focus on both local details and broader global contexts. This results in superior performance in preserving vessel continuity, as shown in rows 6 and 8, where VDMNet exhibits fewer discontinuities or breaks in the vessels compared to the other models. Discontinuities in segmented vessels can lead to incomplete or fragmented vascular structures, which compromise segmentation accuracy, especially in disease diagnosis. This ability to balance fine vessel segmentation with global connectivity is a significant advantage of VDMNet.

Another challenging aspect of retinal vessel segmentation is handling curved and crossing vessels. In rows 6 and 8 of Figure 4, models like UNet3+ and UTNet struggle to maintain the continuity of these complex structures, often leading to discontinuities. However, VDMNet’s VDConv adapts dynamically to varying vessel shapes, resulting in a more accurate segmentation of both curved and crossing vessels, particularly in challenging regions like bifurcations.

OCTA images often contain noise and artifacts, which can hinder accurate vessel segmentation. VDMNet, through the use of WAFT Loss, minimizes the impact of noise by balancing false positives and false negatives more effectively. This enables VDMNet to better preserve vessel continuity, particularly for small vessels, while reducing false positives, as shown in the comparison with models like SwinUNet, which, as a transformer-based network, does not perform particularly well in this type of segmentation.

In summary, VDMNet outperforms other models by preserving fine vessel details, maintaining global vascular structures, and accurately handling complex vessel morphologies. The integration of FastMHSA, VDConv, MSF, and WAFT Loss allows VDMNet to achieve more accurate and reliable retinal vessel segmentation, as evidenced by its strong performance on both the ROSE-1 and OCTA-3M datasets.

## 4. Discussion

In this study, we proposed VDMNet, a novel retinal vessel segmentation network designed to address key challenges in OCTA image segmentation, such as noise, class imbalance, and complex vascular morphologies. VDMNet integrates four key components—FastMHSA, VDConv, MSF, and WAFT Loss—which collectively enhance the model’s ability to accurately segment retinal vessels across different scales and morphological complexities.

One of the primary challenges in retinal vessel segmentation is the accurate identification of small, fine vessels, which are often missed by models that lack multi-scale information handling capabilities. The MSF module in VDMNet addresses this issue by fusing features from multiple scales, enabling the model to maintain vessel continuity from large trunks to smaller branches. This capacity was demonstrated through our experiments, where VDMNet consistently outperformed state-of-the-art methods in preserving fine vessel structures, particularly at bifurcations and terminal branches, areas where models such as UNet and UNet3+ struggled.

Additionally, VDConv dynamically adapts to the varying shapes of retinal vessels, capturing both straight and curved vessels with higher accuracy. The model’s superior performance in handling complex vessel morphologies, such as bifurcations and crossings, was evident in comparison with models like SwinUNet, which often exhibited discontinuities in such regions. The inclusion of FastMHSA allowed the model to maintain balance between the global vessel structure and local details, ensuring continuity and connectivity even in complex topologies, where traditional CNN-based models often struggle.

Moreover, WAFT Loss played a critical role in addressing class imbalance, prioritizing vessel detection and reducing false positives, especially in noisy OCTA images. By combining an asymmetric weighting scheme with focal loss characteristics, WAFT Loss proved more effective than traditional loss functions, such as Dice Loss and Tversky Loss, in preserving fine vessel details and maintaining global structural integrity.

While each module addresses specific challenges, these components are not isolated: they synergistically enhance each other’s contributions, forming a cohesive system that improves segmentation performance. This integrated approach allows VDMNet to outperform existing models across multiple metrics, ensuring both local detail retention and global vessel continuity.

Despite these advancements, VDMNet has limitations. The model was trained and tested on a limited number of datasets, which may impact its generalizability to broader clinical applications. Additionally, VDMNet’s relatively high computational complexity may limit its real-time applicability in clinical settings. Future work will focus on optimizing the model’s computational efficiency through techniques such as model quantization, pruning, and knowledge distillation, while also validating its generalizability across more diverse datasets, imaging modalities, and clinical scenarios. Moreover, artifacts and variations in illumination present in OCTA images still pose challenges. More sophisticated noise-handling techniques, such as advanced denoising algorithms or improved preprocessing methods, could further refine the model’s segmentation performance. Expanding VDMNet’s applicability could also involve integrating multi-task learning approaches, where vessel segmentation is coupled with other related medical image processing tasks, such as lesion detection, disease classification based on segmentation results, and multimodal data integration, possibly utilizing clustering techniques to effectively group and analyze the integrated data [46], thereby enabling a more comprehensive diagnostic framework. Furthermore, unsupervised domain adaptation techniques, like CycleGAN, may improve generalization across different OCTA scanners and patient populations. Finally, integrating explainable AI (XAI) methods could improve the model’s transparency [47], offering insights into its decision-making process and enhancing its clinical trustworthiness.

## 5. Conclusions

In this paper, we introduced VDMNet, a novel retinal vessel segmentation network which integrates advanced components such as FastMHSA, VDConv, MSF, and WAFT Loss to tackle key challenges in OCTA image segmentation. Through comprehensive evaluations on the ROSE-1 and OCTA-3M datasets, VDMNet significantly outperformed state-of-the-art methods across metrics such as DICE, JAC, BACC, REC, CAL, and LCC.

Our proposed model successfully balances the extraction of both global and local features, ensuring vessel continuity and accurately handling complex vessel morphologies. The model’s ability to capture fine vessels and maintain vascular integrity while addressing class imbalance issues highlights its robustness and accuracy in retinal vessel segmentation.

## Figures and Tables

**Table 1:** Ablation experiments results on ROSE-1 dataset.

Baseline	FastMHSA	VDConv	Multi-Scale Fusion	DICE	JAC
**✓**				87.63	77.99
**✓**	**✓**			87.93	78.47
**✓**		**✓**		87.87	78.38
**✓**			**✓**	88.57	79.49
**✓**		**✓**	**✓**	88.77	79.82
**✓**	**✓**		**✓**	88.78	79.84
**✓**	**✓**	**✓**		88.15	78.82
**✓**	**✓**	**✓**	**✓**	89.36	80.79

**Table 2:** Loss function comparison on ROSE-1 dataset.

Method	DICE	JAC	BACC	REC	CAL	LCC
DiceLoss	88.67	79.66	94.11	90.42	78.63	71.14
CrossEntropyLoss	88.36	79.18	93.57	89.19	78.37	80.39
TverskyLoss	88.76	79.81	93.55	88.95	79.08	69.02
FocalLoss	88.05	78.68	93.69	89.64	76.91	71.12
AFT Loss	89.06	80.30	93.95	89.81	79.59	79.20
WAFT Loss (ours)	89.36	80.79	94.32	90.63	80.45	79.60

**Table 3:** Comparison results on ROSE-1 dataset.

Method	DICE	JAC	BACC	REC	CAL	LCC
UNet [43]	82.57	70.34	89.49	81.52	66.13	71.53
UNet3+ [44]	87.13	77.23	92.21	86.31	74.69	75.20
VesselNet [45]	86.19	75.75	93.18	89.26	73.31	75.37
TransUNet [31]	84.61	73.36	90.67	83.51	69.32	81.00
UTNet [39]	87.36	77.58	93.14	88.55	75.94	77.58
SwinUNet [33]	77.06	62.73	86.99	77.85	56.50	57.88
SegFormer [34]	85.09	74.07	91.86	86.39	72.98	80.42
VDMNet (ours)	89.36	80.79	94.32	90.63	80.45	79.60

**Table 4:** Comparison results on OCTA-3M dataset.

Method	DICE	JAC	BACC	REC	CAL	LCC
UNet [38]	90.60	82.88	94.91	-	-	-
UNet3+ [38]	90.92	83.41	94.57	-	-	-
VesselNet [45]	90.90	83.38	94.38	89.27	84.62	90.25
TransUNet [31]	89.89	81.70	94.16	88.94	84.16	88.59
UTNet [39]	91.12	83.76	95.47	91.59	85.10	90.04
SwinUNet [33]	84.42	73.14	90.96	82.84	72.30	65.30
SegFormer [34]	89.25	80.67	93.51	87.63	83.74	82.21
VDMNet (ours)	91.66	84.67	95.43	91.42	87.28	89.21

Missing indicators in the cited literature are represented with ‘-’.

## Data Availability

In our experiment, we utilized two publicly available datasets: ROSE-1 and OCTA-500 (subset OCTA-3M). These datasets can be obtained from the following links: https://imed.nimte.ac.cn/dataofrose.html (accessed on 24 January 2024), https://ieee-dataport.org/open-access/octa-500 (accessed on 22 January 2024).

## References

[1] Antonetti D.A., Klein R., Gardner T.W. (2012). Mechanisms of Disease Diabetic Retinopathy. N. Engl. J. Med..

[2] Bulut M., Kurtulus F., Gözkaya O., Erol M.K., Cengiz A., Akidan M., Yaman A. (2018). Evaluation of optical coherence tomography angiographic findings in Alzheimer’s type dementia. Br. J. Ophthalmol..

[3] Moons L., De Groef L. (2022). Multimodal retinal imaging to detect and understand Alzheimer’s and Parkinson’s disease. Curr. Opin. Neurobiol..

[4] Jia Y., Wei E., Wang X., Zhang X., Morrison J.C., Parikh M., Lombardi L.H., Gattey D.M., Armour R.L., Edmunds B. (2014). Optical Coherence Tomography Angiography of Optic Disc Perfusion in Glaucoma. Ophthalmology.

[5] Spaide R.F., Fujimoto J.G., Waheed N.K. (2015). Image Artifacts in Optical Coherence Angiography. Retin.-J. Retin. Vitr. Dis..

[6] Hormel T.T., Huang D., Jia Y.L. (2021). Artifacts and artifact removal in optical coherence tomographic angiography. Quant. Imaging Med. Surg..

[7] Park J.J., Soetikno B.T., Fawzi A.A. (2016). Characterization of the Middle Capillary Plexus Using Optical Coherence Tomography Angiography in Healthy and Diabetic Eyes. Retin.-J. Retin. Vitr. Dis..

[8] Hanssen H., Streese L., Vilser W. (2022). Retinal vessel diameters and function in cardiovascular risk and disease. Prog. Retin. Eye Res..

[9] Fraz M.M., Remagnino P., Hoppe A., Uyyanonvara B., Rudnicka A.R., Owen C.G., Barman S.A. (2012). Blood vessel segmentation methodologies in retinal images—A survey. Comput. Meth. Programs Biomed..

[10] Orlando J.I., Prokofyeva E., Blaschko M.B. (2017). A Discriminatively Trained Fully Connected Conditional Random Field Model for Blood Vessel Segmentation in Fundus Images. IEEE Trans. Biomed. Eng..

[11] Mendonça A.M., Campilho A. (2006). Segmentation of retinal blood vessels by combining the detection of centerlines and morphological reconstruction. IEEE Trans. Med. Imaging.

[12] Almotiri J., Elleithy K., Elleithy A. (2018). Retinal Vessels Segmentation Techniques and Algorithms: A Survey. Appl. Sci..

[13] Liskowski P., Krawiec K. (2016). Segmenting Retinal Blood Vessels With Deep Neural Networks. IEEE Trans. Med. Imaging.

[14] Mookiah M.R.K., Acharya U.R., Lim C.M., Petznick A., Suri J.S. (2012). Data mining technique for automated diagnosis of glaucoma using higher order spectra and wavelet energy features. Knowl.-Based Syst..

[15] Xie S., Girshick R., Dollár P., Tu Z., He K. Aggregated Residual Transformations for Deep Neural Networks. Proceedings of the 30th IEEE/CVF Conference on Computer Vision and Pattern Recognition (CVPR).

[16] Zhou Z., Siddiquee M.M.R., Tajbakhsh N., Liang J. UNet plus plus: A Nested U-Net Architecture for Medical Image Segmentation. Proceedings of the 4th International Workshop on Deep Learning in Medical Image Analysis (DLMIA)/8th International Workshop on Multimodal Learning for Clinical Decision Support (ML-CDS).

[17] Oktay O., Schlemper J., Folgoc L.L., Lee M., Heinrich M., Misawa K., Mori K., McDonagh S., Hammerla N.Y., Kainz B. (2018). Attention U-Net: Learning Where to Look for the Pancreas. arXiv.

[18] Li X., Chen H., Qi X., Dou Q., Fu C., Heng P. (2018). H-DenseUNet: Hybrid Densely Connected UNet for Liver and Tumor Segmentation From CT Volumes. IEEE Trans. Med. Imaging.

[19] Litjens G., Kooi T., Bejnordi B.E., Setio A.A.A., Ciompi F., Ghafoorian M., van der Laak J., van Ginneken B., Sánchez C.I. (2017). A survey on deep learning in medical image analysis. Med. Image Anal..

[20] Sudre C.H., Li W., Vercauteren T., Ourselin S., Cardoso M.J. Generalised Dice Overlap as a Deep Learning Loss Function for Highly Unbalanced Segmentations. Proceedings of the 3rd MICCAI International Workshop on Deep Learning in Medical Image Analysis (DLMIA)/7th International Workshop on Multimodal Learning for Clinical Decision Support (ML-CDS).

[21] Zhu J., Park T., Isola P., Efros A.A. Unpaired Image-to-Image Translation using Cycle-Consistent Adversarial Networks. Proceedings of the 16th IEEE International Conference on Computer Vision (ICCV).

[22] Cao J., Xu Z., Xu M., Ma Y., Zhao Y. (2023). A two-stage framework for optical coherence tomography angiography image quality improvement. Front. Med..

[23] Chen T., Kornblith S., Norouzi M., Hinton G. A Simple Framework for Contrastive Learning of Visual Representations. Proceedings of the International Conference on Machine Learning (ICML), Electr Network.

[24] Chen Z., Xiong Y., Wei H., Zhao R., Duan X., Shen H. (2022). Dual-consistency semi-supervision combined with self-supervision for vessel segmentation in retinal OCTA images. Biomed. Opt. Express.

[25] Khadka R., Jha D., Hicks S., Thambawita V., Riegler M.A., Ali S., Halvorsen P. (2022). Meta-learning with implicit gradients in a few-shot setting for medical image segmentation. Comput. Biol. Med..

[26] Spaide R.F., Fujimoto J.G., Waheed N.K., Sadda S.R., Staurenghi G. (2018). Optical coherence tomography angiography. Prog. Retin. Eye Res..

[27] Liu Q., Dou Q., Yu L., Heng P. (2020). MS-Net: Multi-Site Network for Improving Prostate Segmentation With Heterogeneous MRI Data. IEEE Trans. Med. Imaging.

[28] Huang K., Yang Y., Huang Z., Liu Y., Lee S. (2023). Retinal Vascular Image Segmentation Using Improved UNet Based on Residual Module. Bioengineering.

[29] Hussain T., Shouno H. (2024). MAGRes-UNet: Improved Medical Image Segmentation Through a Deep Learning Paradigm of Multi-Attention Gated Residual U-Net. IEEE Access.

[30] Yuan X., Huang Y., An L., Qin J., Lan G., Qiu H., Yu B., Jia H., Ren S., Tan H. (2022). Image enhancement of wide-field retinal optical coherence tomography angiography by super-resolution angiogram reconstruction generative adversarial network. Biomed. Signal Process. Control.

[31] Chen J., Lu Y., Yu Q., Luo X., Wang Y., Lu L., Yuille A.L., Zhou Y. (2021). TransUNet: Transformers Make Strong Encoders for Medical Image Segmentation. arXiv.

[32] Liu Z., Lin Y., Cao Y., Hu H., Wei Y., Zhang Z., Lin S., Guo B. Swin Transformer: Hierarchical Vision Transformer using Shifted Windows. Proceedings of the 18th IEEE/CVF International Conference on Computer Vision (ICCV), Electr Network.

[33] Cao H., Wang Y., Chen J., Jiang D., Zhang X., Tian Q., Wang M. Swin-Unet: Unet-like Pure Transformer for Medical Image Segmentation. Proceedings of the European conference on computer vision.

[34] Xie E., Wang W., Yu Z., Anandkumar A., Alvarez J.M., Luo P. SegFormer: Simple and Efficient Design for Semantic Segmentation with Transformers. Proceedings of the 35th Conference on Neural Information Processing Systems (NeurIPS), Electr Network.

[35] He X., Zhou Y., Zhao J., Zhang D., Yao R., Xue Y. (2022). Swin Transformer Embedding UNet for Remote Sensing Image Semantic Segmentation. IEEE Trans. Geosci. Remote Sens..

[36] Ma Y., Hao H., Xie J., Fu H., Zhang J., Yang J., Wang Z., Liu J., Zheng Y., Zhao Y. (2021). ROSE: A Retinal OCT-Angiography Vessel Segmentation Dataset and New Model. IEEE Trans. Med. Imaging.

[37] Li M., Chen Y., Ji Z., Xie K., Yuan S., Chen Q., Li S. (2020). Image Projection Network: 3D to 2D Image Segmentation in OCTA Images. IEEE Trans. Med. Imaging.

[38] Li M., Huang K., Xu Q., Yang J., Zhang Y., Ji Z., Xie K., Yuan S., Liu Q., Chen Q. (2024). OCTA-500: A retinal dataset for optical coherence tomography angiography study. Med. Image Anal..

[39] Gao Y., Zhou M., Metaxas D. UTNet: A Hybrid Transformer Architecture for Medical Image Segmentation. Proceedings of the International Conference on Medical Image Computing and Computer Assisted Intervention (MICCAI), Electr Network.

[40] Ryu J., Rehman M.U., Nizami I.F., Chong K.T. (2023). SegR-Net: A deep learning framework with multi-scale feature fusion for robust retinal vessel segmentation. Comput. Biol. Med..

[41] Giarratano Y., Bianchi E., Gray C., Morris A., MacGillivray T., Dhillon B., Bernabeu M.O. (2020). Automated Segmentation of Optical Coherence Tomography Angiography Images: Benchmark Data and Clinically Relevant Metrics. Transl. Vis. Sci. Technol..

[42] Tan X., Chen X., Meng Q., Shi F., Xiang D., Chen Z., Pan L., Zhu W. (2023). OCT2Former: A retinal OCT-angiography vessel segmentation transformer. Comput. Meth. Programs Biomed..

[43] Ronneberger O., Fischer P., Brox T. U-Net: Convolutional Networks for Biomedical Image Segmentation. Proceedings of the 18th International Conference on Medical Image Computing and Computer-Assisted Intervention (MICCAI).

[44] Huang H., Lin L., Tong R., Hu H., Zhang Q., Iwamoto Y., Han X., Chen Y., Wu J. UNet 3+: A Full-Scale Connected UNet for Medical Image Segmentation. Proceedings of the IEEE International Conference on Acoustics, Speech, and Signal Processing (ICASSP).

[45] Wu Y., Xia Y., Song Y., Zhang D., Liu D., Zhang C., Cai W. Vessel-Net: Retinal Vessel Segmentation Under Multi-path Supervision. Proceedings of the 10th International Workshop on Machine Learning in Medical Imaging (MLMI)/22nd International Conference on Medical Image Computing and Computer-Assisted Intervention (MICCAI).

[46] Hussain I., Sinaga K.P., Yang M.S. (2023). Unsupervised Multiview Fuzzy C-Means Clustering Algorithm. Electronics.

[47] Hussain T., Shouno H. (2023). Explainable Deep Learning Approach for Multi-Class Brain Magnetic Resonance Imaging Tumor Classification and Localization Using Gradient-Weighted Class Activation Mapping. Information.

